# Comparative Evaluation of the Effect of Manufacturing Process on Distortion of Rotary ProFile and Twisted File: An in Vitro SEM Study

**DOI:** 10.15171/joddd.2015.039

**Published:** 2015-12-30

**Authors:** Swati Sharma, Rajendra Kumar Tewari, Pankaj Kharade, Pankaj Kharade

**Affiliations:** ^1^Senior Resident, Masters in Dental Surgery, Department of Conservative Dentistry and Endodontics, Dr. Z.A. Dental College, Aligarh Muslim University, Aligarh, India; ^2^Professor, Department of Conservative Dentistry and Endodontics, Dr. Z.A. Dental College, Aligarh Muslim University, Aligarh, India; ^3^Assistant Professor, Department of Prosthodontics, Dr. Z.A. Dental College, Aligarh Muslim University, Aligarh, India

**Keywords:** Distortion, Ni-Ti files, ProFile, Scanning electron microscope, Twisted file

## Abstract

***Background and aims.*** The manufacturing process of rotary Ni-Ti file can influence its resistance to fracture. The rotary ProFile (Dentsply-Maillefer, Baillagues, Switzerland) is manufactured by grinding mechanism whereas Twisted File (Sy-bron Endo, USA) is manufactured with a twisting method. The purpose of this study was to comparatively evaluate the effect of manufacturing process on distortion of rotary ProFile and Twisted files using scanning electron microscopy after in vitro use.

***Materials and methods.*** Five sets of each type of file were used for this study -rotary ProFile (group A) and Twisted file (group B). Each set was used according to manufacturer’s instructions to prepare 5 mesial canals of extracted mandibular molars. The changes in files were observed under a scanning electron microscope at ×18, ×100, ×250 and ×500 magnifications. Observations were classified as intact with no discernible distortion, intact but with unwinding, and fractured. Group A and B were then compared for deformation and fracture using two-proportion z-test.

***Results.*** On SEM observation, used rotary ProFile showed microfractures along the machining grooves whereas Twisted file showed crack propagation that was perpendicular to the machining marks. On statistical analysis, no significant difference was found between ProFile and Twisted file for deformation (P=0.642) and fracture (P=0.475).

***Conclusion.*** Within the experimental protocol of this study, it was concluded that both ProFile and Twisted files exhibited visible sign of distortion before fracture. But Twisted file gained edge over ProFile because of its manufacture design and unparalleled resistance to breakage.

## Introduction

Over the years, various scanning electron microscopic (SEM) studies have been carried out to evaluate distortion of nickel-titanium endodontic instruments after in vitro and in vivo use. These studies have contributed towards understanding the changes in structure of these Ni-Ti endodontic instruments at a microscopic level.^1-3^

Numerous factors such as the operator proficiency, method of use, rotational speed, anatomic configuration of the canals, design of the instrument, and number of sterilization cycles have been implicated in the separation of Ni-Ti endodontic instrument.^4-6^Manufacturing process of rotary Ni-Ti file can also influence the distortion of Ni-Ti endodontic instrument.^7,8^These distortions can be in the form of unwinding of flutes, microcracks, pitting or surface wear. In order to avoid instrument fracture, it is important to check the instruments for signs of wear and deformation every time it is used.

Since the introduction of nickel-titanium alloy in endodontics, there have been many changes in instrument design, but no significant improvements in the raw material properties, or enhancements in the manufacturing process could be achieved. Traditional rotary Ni-Ti files, such as Profile, are manufactured by grinding process. This grinding mechanism can produce microcracks and areas of metal rollover on the cutting flutes of the file that are the focus of subsequent file fracture if the file is subjected to excessive torsion and cyclic fatigue.^9^

On the contrary, Twisted file is manufactured by twisting of the metal. The twisting of a ground blank of metal in combination with heat treatment enhances super-elasticity and increases resistance to cyclic fatigue.^10^

The purpose of this study was to comparatively evaluate the distortion of rotary ProFile and Twisted files, which are manufactured by different manufacturing processes, using scanning electron microscopy after in vitro use.

## Materials and Methods

This study consisted of two groups of files - ProFile (group A) and Twisted file (group B). Each group consisted of 5 sets of files. Fifty mesial canals of extracted mandibular first and second molars with a canal curvature between 20° and 40°were then selected for the study. The canal curvature was calculated by Image J software (Wayne Rasband; National Institutes of Health, Bethesda, Maryland, USA) using the Schneider technique. Each file set in each group was used to prepare 5 mesial canals which were randomly assigned to each group. All the instrumentation procedures were performed according to each manufacturer’s instructions. After access cavity preparation, straight-line access was first achieved with #10 K-file and working length was determined. The apical portion of canal was then enlarged to #15 K-file to establish an apical glide path. The ProFile (Dentsply- Maillefer, Baillagues, Switzerland) instruments of following sizes (.06 – 30, .06 – 25, .06 – 20, .04 – 30, .04 – 25, and .04 – 20) were selected for this study. ProFiles were used at 300 rpm. These settings were within the range suggested by the manufacturer. Small configuration (25 tip size with taper of .04, .06, and .08) of 23-mm length of Twisted files was used for this study as indicated for mesial roots of mandibular molars. Twisted files were used at 500 rpm as recommended by the manufacturer.

After each use, the instruments were wiped with a piece of gauze soaked with isopropyl alcohol and inspected under ×2.5 magnification for signs of fracture and flute distortion. Instruments were discarded when they had reached the designated number of uses, or when they were worn, fractured, or had any other defects.

Before observation under SEM, all the used files were wiped with an alcohol-soaked piece of gauze and subsequently cleaned in an ultrasonic cleaner to completely eliminate any residues that remained attached to the surface of the blades and finally it was autoclaved. The handles of the files were then cut off from the used file samples as well as the unused control group, using a slow-speed water-cooled diamond saw. The remaining portion of file was then mounted on metallic stubs and gold-sputtered in order to make the surface conductive for SEM evaluation. The samples were then viewed under a scanning electron microscope at various magnifications. After general survey scan of each file at a magnification of ×18, an image of the most representative area of the file was taken at ×100, ×250 and ×500 magnifications. The defects observed consisted of pits or cracks, unwinding of file, blunting (rolling-over) of the cutting edges, surface debris and fracture. Any defect or distortion (plastic deformation) noted was classified into one of the following categories:

(1) intact with no discernible distortion or unwinding

(2) intact but with unwinding defects

(3) fractured

Data was analyzed statistically using two-proportion z-test and comparisons were made between the two groups (A and B) for deformation and fracture.

## Results

**Figure 1.**
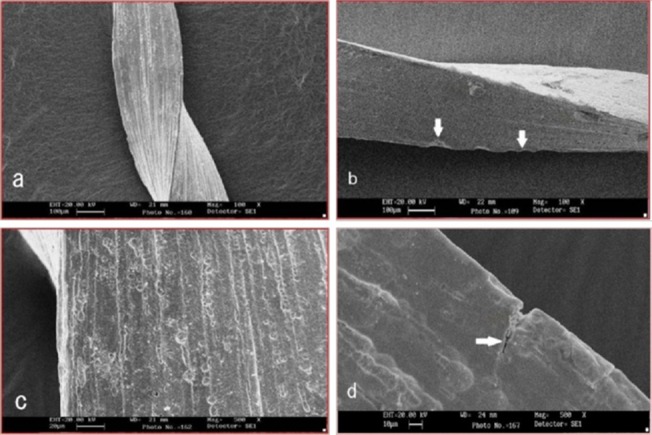
Scanning electron microscopic images of ProFile. (a) Unused ProFile at ×100. (b) Used ProFile at ×100, showing defects (metal roll over). (c) Unused ProFile at ×500. (d) Used ProFile at ×500, showing cracks along machining grooves

**Figure 2.**
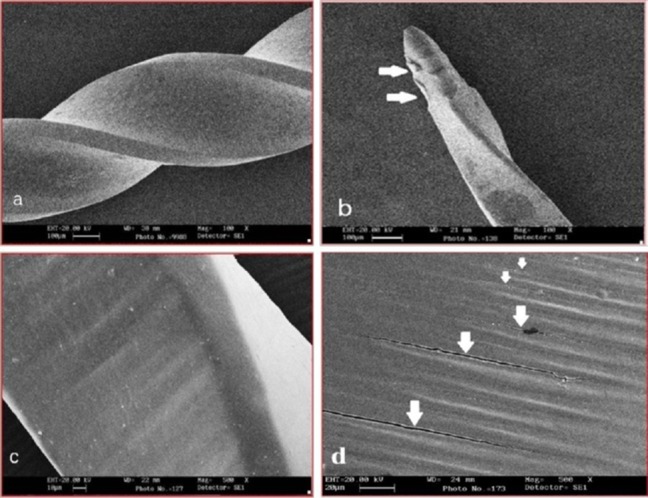
Scanning electron microscopic images of Twisted file. (a) Unused Twisted file at ×100. (b) Used Twisted file at ×100, showing defects (disruption of cutting edges). (c) Unused Twisted file at ×500. (d) Used Twisted file at ×500, showing cracks with debris inside

On SEM observation of control groups, unused ProFile showed machining marks that ran transverse to the long axis of the instrument (Figure 1a,c) whereas in case of unused twisted files they were along the long axis of the instrument (Figure 2a,c).

On SEM observation of files after instrumentation, microfractures along the machining grooves were noted for rotary ProFile (Figure 1b,d) whereas Twisted file showed crack propagation that was not related to the machining grooves and in a few samples they were found perpendicular to the machining marks (Figure 2b,d).

On Intergroup comparisons for fracture, Twisted file (0/15 or 0%) showed no fracture as compared to ProFile (1/30 or 3.33%). However, statistically no significant difference for fracture was found between group A (ProFile) and group B (Twisted file) (P = 0.475). For ProFile, fractured instrument was of size 0.04, 20.

On Intergroup comparison for deformation, twisted file (5/15 or 33.33%) showed higher deformation (unwound) than ProFile (8/30 or 26.7%). However, statistically no significant difference was found between group A (ProFile) and group B (Twisted file) for deformation (P=0.642). In case of ProFile, visible deformations were found only in instruments of #25 or smaller and ProFile of taper 0.04 (6/8) showed the highest visible deformation, whereas Twisted file of #0.04, 25 only showed the visible deformation (5/15).

## Discussion

The aim of this study was to comparatively evaluate the effect of manufacturing process of ProFile and Twisted files on their distortion after in vitro use.

Since the introduction of nickel-titanium alloy to endodontics, there have been no significant improvements in the raw material properties, or enhancements in the manufacturing processes. The ProFile system is one of the first Ni-Ti instruments marketed, whereas Twisted file is a new introduction. Traditional rotary Ni-Ti files (ProFile) are manufactured by grinding process. Grinding during manufacture can produce microcracks and areas of metal rollover on the cutting flutes of the file. Microcracks and manufacturing defects are the focus of subsequent file fracture if the file is subjected to excessive torsion and cyclic fatigue.^9^This can be observed in this study on scanning electron microscopic images of used ProFile which is manufactured by grinding process. Microfractures along the machining grooves were seen on SEM observation of used ProFile in this study.

Recently, a new manufacturing process was developed to create Twisted file, which enhances its super-elasticity and increases cyclic fatigue resistance.^11^Twisted nickel-titanium files are created by taking the raw nickel titanium wire in the austenite crystalline structure and transforming it into a different phase of crystalline structure (R-phase) by a process of heating and cooling. In R-phase, nickel titanium cannot be ground, but it can be twisted. In this study, on SEM observation Twisted file showed crack propagation on SEM images that was not related to the machining marks.

Initiation of fatigue crack usually occurs if the areas with highest stress coincide with the machining marks or miniature grooves.^12^ Thus, electropolishing was tried to remove the machining scratch marks to enhance the (fatigue) fracture resistance.^13^ In this study, ProFile system used is non-electropolished whereas Twisted file is electropolished.

Stewart et al^14^in their in vitro study on ProFile reported a distortion rate of 19.4% (7/36) and a fracture rate of 2.8% (1/36). Ankrum et al^15^ reported a distortion rate of 15.3% (nine out of 59 files) and Yancy et al^16^ reported a 9.5% distortion rate of ProFile without any file separation. In our study, the deformation rate of 26.7% (8/30) and fracture rate of 3.33% (1/30) was reported for ProFile group. The variation in results can be explained on the basis of variations in experimental designs such as the number of times the instrument was used, curvature of the canal, and rotational speed of the ProFile as these may affect the distortion rate of ProFile. In this study, the visible distortion in ProFile was reported in the form of unwinding of flutes, which is consistent with the findings of previous studies.^14,15^

Sattapan et al^2^ reported that the mode of separation of NiTi rotary instruments may be classified into flexural fatigue and torsional (shear) fracture. Torsional fracture results when the instrument exceeds the elastic limit of the metal, producing plastic deformation followed by fracture. This occurs when the tip or any part of the instrument binds in the canal and rotary motion continues. Flexural fracture occurs because of metal fatigue. This happens at the point of canal curvature, when the instrument is freely rotating. In this study, the smallest ProFile instrument (.04, #20) demonstrated fracture, which is consistent with the findings of some previous studies.^17,18^Instruments of #20 when used for apical enlargement and in crown-down technique there is more engagement of smaller instruments close to their tips. Therefore, torsional fracture might be the cause of separated ProFile in this study as fracture occurred near the tip of the instrument.

To date, only very few reports of new twisted Ni-Ti files are available.,^7,19,20^ In this study Twisted file only exhibited deformation but no fracture, which is consistent with the findings of a study by Caballero et al.^21^ The deformation was observed only in 0.04 taper file in the form of lengthening of flutes (unwinding of flutes). The percentage of deformed Twisted file in this study was 33.33% (5/15). These results can be explained on the basis of manufacturing process of Twisted file. The manufacturer claimed that TF has a different surface texture (natural grain structure) that runs longitudinally and that the instrument is made of the R-phase of Ni-Ti alloy. These features raise the flexibility and the fracture resistance of the instrument.^22,23^There is also an absence of transverse-running machining marks (as a result of electropolishing) that would result in slower crack initiation and propagation.

ProFile and Twisted file systems have shown visible signs of distortion in the form of unwinding before fracture. These findings lead us to the fact that these instruments should be used carefully and in the prescribed manner only. They should be checked visually under magnification after each use and the distorted ones should be discarded.

However, statistically no significant difference (P>0.05) was found between the fracture rate and deformation rate of Group A (ProFile system) and Group B (Twisted file) in this study, which is consistent with the findings of some other studies.^7^ Twisted file has an advantage over ProFile system because of its manufacture design and unparalleled resistance to breakage.

Apart from manufacturing process, there are other variables also that may affect the performance of rotary endodontic instruments in clinical practice and their resistance to fatigue and separation. Some of these variables are size, taper and sequence of files along with the operator’s skill.^24,25^Therefore, within the limitations of this study, it is important for clinicians to know the characteristics of different file designs and associated implications for use in different clinical situations. Each file performs better in some areas and worse in others and this information is important to help choose the best instruments for each clinical case.

## Conclusion

ProFile and Twisted file systems showed visible signs of distortion in the form of unwinding before fracture. These finding suggest that these instruments should be used carefully according to the manufacturer’s instructions and should be checked visually under magnification for any signs of deformation which alert the clinician to discard the deformed instrument to prevent a possible intracanal fracture.
